# Advances in the management of gallbladder polyps: establishment of predictive models and the rise of gallbladder-preserving polypectomy procedures

**DOI:** 10.1186/s12876-023-03094-7

**Published:** 2024-01-02

**Authors:** Haoran Liu, Yongda Lu, Kanger Shen, Ming Zhou, Xiaozhe Mao, Rui Li

**Affiliations:** Department of Gastroenterology, The First Affiliated Hospital of Soochow University, Pinghai Road, Gusu District, Suzhou, 215000 Jiangsu China

**Keywords:** Gallbladder polyp, Gallbladder cancer, Predictive models, Gallbladder-preserving polypectomy procedures, Minimally invasive Surgery

## Abstract

Gallbladder polyps are a common biliary tract disease whose treatment options have yet to be fully established. The indication of “polyps ≥ 10 mm in diameter” for cholecystectomy increases the possibility of gallbladder excision due to benign polyps. Compared to enumeration of risk factors in clinical guidelines, predictive models based on statistical methods and artificial intelligence provide a more intuitive representation of the malignancy degree of gallbladder polyps. Minimally invasive gallbladder-preserving polypectomy procedures, as a combination of checking and therapeutic approaches that allow for eradication of lesions and preservation of a functional gallbladder at the same time, have been shown to maximize the benefits to patients with benign polyps. Despite the reported good outcomes of predictive models and gallbladder-preserving polypectomy procedures, the studies were associated with various limitations, including small sample sizes, insufficient data types, and unknown long-term efficacy, thereby enhancing the need for multicenter and large-scale clinical studies. In conclusion, the emergence of predictive models and minimally invasive gallbladder-preserving polypectomy procedures has signaled an ever increasing attention to the role of the gallbladder and clinical management of gallbladder polyps.

## Background

Gallbladder polyps (GBPs) are elevated lesions of gallbladder mucosa that protrude into the gallbladder lumen. Based on regional and ethnic differences, the prevalence of GBPs varies from 0.3 to 9.5% [1]. Pathologically, GBPs can be divided into non-neoplastic polyps, including cholesterol polyps, gallbladder adenomyosis and inflammatory polyps, as well as neoplastic polyps, which refer to adenomas and adenocarcinomas. Most GBP patients are asymptomatic and are usually found incidentally on imaging or pathological examination after cholecystectomy. A small percentage of patients develop acute cholecystitis due to polyp obstruction of the cystic duct, or cholangitis due to polyp rupture with the fragments descending into the bile duct [2].

In clinical practice, it is often considered that a GBP with a diameter ≥ 10 mm is likely to be malignant; even if it is not malignant, it is more likely to be an adenoma with malignancy potential. In addition, gallbladder cancer (GBC) is an aggressive malignant tumor with poor prognostic outcomes. Therefore, clinicians always adopt aggressive therapeutic options for such GBP patients. However, Lee et al. found that the incidence of neoplastic polyps in patients undergoing cholecystectomy for GBPs was only 24.0% [3], implying that over 70% of GBP patients have had their gallbladders removed because of benign polypoid lesions. In a cohort study of the relationship between GBPs and GBC conducted in the general Northern California population, 99.6% (2,047 of 2,055 in the study) of polyps that were 10 mm or larger in size were non-neoplastic [4].

Given that cholecystectomy is associated with various complications, such as bile duct injury and bile leakage, and that gallbladder absence increases the rate of enterohepatic recirculation of bile acids, inducing metabolism-related adverse effects, and the risk of non-alcoholic fatty liver, cirrhosis, and small bowel carcinoid [5], the decision to remove the gallbladder has become increasingly cautious in recent years. Establishment of predictive models using statistical tools and artificial intelligence (AI), as well as the rise of minimally invasive gallbladder-preserving polypectomy procedures that are able to eradicate lesions while retaining a functional gallbladder to maximize the benefit to patients with non-neoplastic GBPs, has promoted the identification and treatment of benign and malignant polyps. This article focuses on the progress of predictive models in the past decade and gallbladder-preserving polypectomy procedures for GBPs.

## Predictive models

In the last decade, with advances in medical statistics and statistical tools, the risk factors provided solely by the guidelines cannot intuitively show the malignant potential of GBPs. Therefore, many predictive models have been built using conventional clinical data, such as data from laboratory tests, imaging results, pathological specimens and bile acids, combined with decision tree, nomogram, scoring model, Bayesian network and some other statistical tools. (Table 1) As the earliest reported predictive model, Yang’s Model [6], established on the basis of patient age and ultrasound findings, has good sensitivity and specificity in diagnosing potentially malignant GBPs. Kim E combined bile viscosity and bile cholesterol with patient age to intuitively design a predictive scoring model [7]. The procedures for obtaining bile acids in clinical use are invasive, however, to some extent, the model is able to avoid cholecystectomy for cholesterol polyps, in light of its good performance in differentiating adenomatous from cholesterol polyps. Unlike other studies that focused only on a single diameter, Ma first introduced the concept of cross-section area (CSA), which is calculated by multiplying the two longest diameters of the polyp provided in the ultrasound report, and validated that a CSA > 123 mm2 was the most significant risk factor in their predictive scoring model [8].

**Table 1:** Predictive models based on conventional clinical data in the last decade

Name	Published Time	Major Tool	Relevant Factors	Evaluation
Yang’s Model(6)	2018-03	Ultrasound	✓ Age ✓ Number (single/ multiple) ✓ Sessile/ pedunculated ✓ Polyp size	✓ Predictive score (PS) = − 7.3633 + 0.0374 × [Age] + 0.6667 × [Number] + 1.5784 × [Sessile] + 0.2189 × [Size]. Probability of neoplastic polyp = e^PS^ / (1 + e^PS^), where e = 2.7182. ✓ The AUC was 0.83 and 0.90 in the modeling group and validation group, respectively. When the cut-off value of the neoplastic probability was 7.4%, the sensitivity of the model was 78.5% and the specificity was 77.5%. In the entire cohort, only 2 individuals (6.7%) with malignant polyp were missed with this cut-off value. ✓ It proved the model could be useful in clinical practice to predict neoplastic potential of gallbladder polyp more accurately than only considering each risk factor of neoplastic gallbladder polyp.
Wennmacker’s Model [9]	2018-09	Pathology report	✓ Polyp size ✓ Number ✓ Wall thickening ✓ Protruding polyp ✓ Presence of gallstones	✓ The decision tree using the surgical threshold data and clinicopathological characteristics of neoplastic and non-neoplastic polyps was established, which results in the prediction for each of the 16 possible combinations of clinicopathological characteristics. ✓ The AUC was 0.75. The curve showed that 1 cm is the most optimal size threshold for differentiating neoplastic and non-neoplastic polyps. Sensitivity of the surgical threshold for indicating neoplastic polyps was 68.1% and specificity was 70.2%.
Chen’s Model [13]	2019-10	Contrast-enhanced computed tomography	✓ Size ✓ Stone ✓ Mucosal smoothness (smooth/ irregular) ✓ Layered pattern of gallbladder wall on portal vein phase ✓ Gallbladder wall enhanced ✓ ∆CT value of mass (portal phase – delayed phase) ✓ Age ✓ CA199	✓ The nomogram was established by 6 radiological features and 2 clinical factors. ✓ The AUC in the internal and external validation cohorts were up to 0.91 and 0.89, respectively. it also demonstrated superior sensitivity (95.6%) and accuracy (95.2%) in the diagnosis of GBC in the training cohort. Most of the gallbladder polyps, which were misdiagnosed as benign lesions, were successfully identified using this nomogram. ✓ The nomogram added significant strength for early detection of malignancy in the gallbladder, especially for T1-2 tumors.
Kim E’s Model [7]	2020-08	Bile	✓ Age ✓ Bile viscosity ✓ Bile cholesterol	✓ A predictive scoring model was developed for polypoid lesions of the gallbladder larger than 1 cm to distinguish adenomatous polyps from cholesterol ones. ✓ The AUC was 0.845. The model had a sensitivity of 90.9% and a specificity of 80.2% at a cutoff of ≥ 6 points. The performance of the model was superior to Wennmacker’s Model [7]. ✓ The process of obtaining bile acids is invasive. Moreover, this study only explored the differences between adenoma and cholesterol polyps, but did not explore the differences between other non-cholesterol benign polyps such as hyperplastic polyps and inflammatory polyps.
Zhang D’s Model [10]	2021-06	Ultrasound	✓ Number of polyps ✓ Fundus (pedicle/ broad base) ✓ Echogenicity ✓ Polyp size (long diameter) ✓ Polyp size (short diameter)	✓ The nomogram prediction model for gallbladder polyps with malignant tendency with a long diameter of 10–15 mm was constructed, which is available at https://docliqi.shinyapps.io/dynnom/. ✓ The consistency index of the model was 0.778 and the internal validation was 0.768.
Zhang X’s 2021 Model [43]	2021-06	Ultrasound	✓ Age ✓ Cholelithiasis ✓ CEA ✓ Polyp size ✓ Sessile	✓ The formula for neoplastic risk in patients with gallbladder polyps was: Y = 1.194 × [age] + 1.177 × [cholelithiasis] + 1.171 × [CEA] + 1.112 × [polyp size] + 1.066 × [sessile] − 3.944. ✓ The AUC was 0.846 and 0.835 in the training and validation cohorts, respectively. The nomogram achieved an overall accuracy rate of 84.1%, with a sensitivity of 68.1% and a specificity of 88.2%. ✓ Compared with Yang’s model(6) and three different management guidelines(JSHBPS, ESGAR and CCBS) at that time, the nomogram achieved significantly better diagnostic performance and provided more clinical benefit.
Ma’s Model [8]	2022-01	Ultrasound	✓ Cross-sectional area ✓ Positive blood flow ✓ Age ✓ ALT ✓ ALT/AST	✓ The scoring model for predicting true polyps was established, and a new reference parameter, the cross-sectional area of a gallbladder polyp, was innovatively introduced. ✓ The AUC was 0.883. A total score of 6.5 was the optimal cut-off value for distinguishing between true polyps and pseudo-polyps, with a sensitivity of 72.7% and a specificity of 89.6% in the reference group.
Zhang X’s 2022 Model [14]	2022-03	Ultrasound	✓ Age ✓ Polyp size ✓ CEA ✓ Gallstone ✓ Sessile shape	✓ The formula for the prediction model was: Y = 1.084 × [age] + 0.937 × [polyp size] + 1.465 × [CEA] + 0.927 × [gallstone] + 0.862 × [sessile] – 4.236. ✓ The nomogram achieved an overall accuracy rate of 86.3% with a sensitivity of 69.5%, a specificity of 90.7%. The model yielded the AUC of 0.845 in the validation cohort. ✓ The model showed better diagnostic performance than Yang’s model(6) and three guidelines(JSHBPS, ESGAR and CCBS) at that time. ✓ Limited by the number of ultrasound images, the minimum caliper diameter was not obtained in the model, despite the research proved that it was the important independent predictor for malignant gallbladder polypoid lesions.
Liu’s Model [12]	2022-06	Ultrasound	✓ Number of polyps ✓ Maximal diameter ✓ Shape (irregular/regular)	✓ The regression equation was logit(P) = -3.828 + 1.083 × number of GPLs + 0.218 × diameter of GPLs + 1.714 × shape of GPLs. ✓ AUC was 0.828. When logit P > 0.204, the sensitivity of estimating adenomatous polyps was 79.5%, the specificity was 70.6% and the whole correct ratio was 73.3%. ✓ The model reduced confounding factors in diagnosing adenomas, and its prediction efficiency is better than Wennmacker’s Model(7).
Li’s Model [11]	2022-08	Ultrasound	✓ Age ✓ Number of polyps ✓ Polyp size (long diameter) ✓ Polyp size (short diameter) ✓ Fundus	✓ A Bayesian network prediction model was available at https://simulator.bayesialab.com/#!simulator/204709691197. ✓ The AUC was 77.38% and 75.13%, and the model accuracy was 75.58% and 80.47% for the Bayesian network model in the training set and testing set, respectively. ✓ The model was accurate and practical for predicting gallbladder polyps with malignant potential patients in a long diameter of 8-15 mm. ✓ The model took not only the long diameter of polyp size, but also the short diameter into consideration.

AUC, area under curve; CA199, carbohydrate antigen199; GBC, gallbladder cancer; CEA, carcinoembryonic antigen; JSHBPS, the Japanese Society of Hepato-Biliary-Pancreatic Surgery; ESGAR, European Society of Gastrointestinal and Abdominal Radiology; CCBS, Chinese Committee of Biliary Surgeons; ALT, alanine aminotransferase; AST, aspartate aminotransferase

The predictive models have yielded good outcomes, however, they have some limitations: First, all studies in the table are retrospective, and the involved patients were those with postoperative pathological results. Some patients with GBPs that were obviously malignant, had infiltrated nearby organs or had low malignancy potential and were selected for follow-up observation were excluded, indicating a selection bias. Kim E attempted to mitigate the selection bias by prospectively sampling and collecting data on every patient who had surgery due to GBPs [7]. Second, most of the studies were single-center, with only 3 multicenter clinical studies, among which the study by Wennmacker [9] was national, while studies by Zhang D [10] and Li [11] were territorial. Single-center studies are not sufficient for establishing accurate predictive models. Besides, some studies [7, 9, 12] did not design validation or testing cohorts, and all studies, apart from that by Chen [13], did not have an external cohort to validate the accuracy of the models, leaving the models’ diagnostic reliability in need of further proof. Finally, given sample size limitations and the inherent nature of retrospective studies, the types of data contained in the model were small, with Asian ethnicity and polyp growth rate, which some guidelines consider as risk factors, not being included in the model. In Zhang X’s study [14], the minimum caliper diameter was not added to the model because of the small sample size, even though it was verified to be an important independent predictor of malignancy. This reinforces the need to conduct multicenter and large-scale clinical trials.

The field of radiomic technology based on AI has rapidly developed in recent years, with computers processing massive datasets through layered mathematical models that can detect patterns that are not otherwise decipherable using biostatistics [15]. By extracting radiomic features from transabdominal contrast-enhanced computed tomography (CT) images of patients with preoperative polyps ≥ 1 cm in length, combined with clinical features of these patients, Yang et al. successfully constructed a diagnostic model that can effectively preoperatively predict and identify the benign and malignant GBPs [16]. A deep learning-based decision support system on ultrasonography exhibited a higher specificity than all human reviewers, especially when the polyp was ≥ 10 mm [17]. The system can improve the performance of a less-experienced radiologist, narrow the gap between reviewers and avoid unnecessary cholecystectomy. Kim T established an ensemble model incorporating three convolutional neural network models (ResNet, Inception v3 and DenseNet), whose diagnostic performance was improved by adding information such as age and polyp size [18]. The improved ensemble model achieved a specificity of 88.35%, AUC of 0.9082, and accuracy of 87.61%, implying a great performance in classifying GBPs of < 20 mm, especially for those > 10 mm. The models built by Yuan et al. achieved good results in identifying the nature of GBPs [19, 20]. Han integrated Radscore, which is calculated by taking 6 radiomics factors into the radiomics model, into the nomogram [21]. Even though the combined model performed slightly worse than the only clinical model in the testing cohort, it was a promising attempt.

The radiomic features-based diagnostic models are also associated with various limitations. On one hand, all of the above mentioned five studies were retrospective in nature. Apart from the study by Kim T [18], whose data were from two territorial hospitals, the other researches were single-center, limited by the small sample size. Multicenter clinical trials with large sample sizes should be conducted to validate the clinical utility of the model. Moreover, to improve the model and make it more practically applicable in clinical practice, there is a need to develop an algorithm that distinguishes the features of polyps in real-time videos instead of still images [18], which provides a direction for future exploration in this field.

## Minimally invasive gallbladder-preserving polypectomy procedures

The above predictive models and AI have had satisfying results in predicting the malignancy of GBPs. Minimally invasive gallbladder-preserving polypectomy procedures are emerging and they can obtain accurate pathological results while preserving gallbladder functions. These methods include percutaneous transhepatic cholecystoscopy (PTCS), percutaneous cholecystoscopic polypectomy (PCP), endoscopic-laparoscopic (Endolap) polypectomy, natural orifice transluminal endoscopic gallbladder-preserving polypectomy (NOTEGPP), embryonic-natural orifice transumbilical endoscopic gallbladder-preserving polypectomy (E-NOTEGPP) and endoscopic ultrasound-guided cholecystostomy (EUS-GC) combined with per-oral transmural endoscopic polypectomy (PTEP).

### PTCS

PTCS is a minimally invasive treatment approach that is based on percutaneous transhepatic cholecystocentesis to dilate the sinus to accommodate a fiberoptic endoscope, which is then used to inspect the gallbladder and excise the GBPs. Initially, this method was used in gallstone treatment, however, in the 1980s, Inui et al. successfully used it to diagnose gallbladder polypoid lesions and to treat benign tumors [22]. PTCS was performed in 72 patients with gallbladder disease, 5 of whom underwent polypectomy. The article describes the complications of PTCS solely in general terms. Five of the 72 patients had catheter dislodgement between two and seven days postoperatively, and three patients had sinus tracts between the liver and the abdominal wall damaged during the operation. There were no reports of serious complications such as bleeding or cholestatic peritonitis. This therapeutic approach is limited by the long treatment period, trauma to the liver, inapplicability to the floating gallbladder and some other disadvantages. The use of PTCS decreased with popularity of laparoscopic cholecystectomy (LC) and the rise of other types of gallbladder-preserving polypectomy procedures.

To shorten the treatment period, there has been an attempt to use smaller sheaths and fluoroscopy for gallstones. Compared with the sinus channel of 18-Fr, the 12-Fr sheath can reduce the hospitalization duration from at least 17 days to 7.3 days (acute cholecystitis) and 9.4 days (gallbladder empyema) [23]. A smaller sheath reduces hepatic hemorrhage complications and pain, however, conversion from direct endoscopic view to fluoroscopic view limits the manipulation and surgical sights, which may affect the use of this method in GBP treatment.

### PCP

PCP, which is also known as minilaparotomy choledochoscopic gallbladder preserving polypectomy (M-CGPP) [24], is an improvement of PTCS. In this operation, a small incision is made under the costal margin of the right upper abdomen after localization by ultrasound. The gallbladder is dragged out of the abdominal cavity by forceps, its fundus is incised and a choledochoscope inserted. In 1994, Ji et al. performed PCP in 5 patients with cholesterol GBPs and 1 patient with adenomatous GBPs [25]. The procedures were successfully performed in all patients, and no complications occurred. After 8–16 months of follow-up, all patients were recurrent-free and gallbladder functions remained good.

The latest data released by researchers in 2004 revealed that for 85 cases, 78.8% of the patients who underwent PCP were followed up for over 5.5 years on average, and 95% of the patients retained good gallbladder functions without gallstone formation and polyp recurrence [26]. The other 3 patients had mild upper abdominal discomfort who proved to have an underfunctioning gallbladder with additional gallstone formation. Ultimately, they had their gallbladders removed laparoscopically. Key points of this procedure are that postoperative gallbladder drainage is not required and only absorbable sutures are needed for closure of the incision. Follow-up revealed that gallbladder drainage led to stone formation and impaired gallbladder motility.

### Endolap polypectomy

Endolap polypectomy, also referred to as laparoscopy-assisted choledochoscopic gallbladder preserving polypectomy (La-CGPP) [24], combines laparoscopy and choledochoscopy to treat GBPs. It is suitable for thin and small patients or patients with drooping and floating gallbladder, which has no adhesion around it. After finding the gallbladder under the laparoscope, its fundus is incised and the choledochoscope is inserted into the gallbladder to treat the polypoid lesions. Wang performed Endolap polypectomy on 60 patients with GBPs and achieved a success rate of 93.33%. Four cases failed to preserve the gallbladder and were converted to LC, including diffuse cholesterosis in three cases and massive adhesions around the gallbladder in one case. Postoperatively, all gallbladder-preserving patients achieved symptomatic relief and no recurrence [27].

A retrospective study comparing the clinical efficacy of Endolap polypectomy and LC in the treatment of gallbladder polypoid lesions found that the gallbladder-preserving procedure could reduce the incidence of complications such as indigestion, diarrhea, and gastroesophageal reflux in patients while achieving satisfactory treatment results [28]. After a 3-year follow-up period, three of the patients (3.84%) with gallbladder preservation were found to have recurring polyps, and two (2.56%) developed cholesterol crystal. As well, by analyzing the results of a five-year single-center cohort study, Tian et al. suggest that Endolap polypectomy could be an alternative management strategy for a group of patients who meet the selection criteria [29].

### NOTEGPP

NOTEGPP is an operation in which GBPs are removed by endoscopy (gastroscopy or colonoscopy) to preserve the gallbladder after artificially penetrating the viscera into the abdominal cavity through the natural human body orifice. Based on approaches, NOTEGPP can be divided into the following two types: (i) Transgastric gallbladder-preserving polypectomy (TG-GPP): The endoscope is inserted into the gastric cavity through the mouth, pharynx and esophagus, after which the gastric cavity is fully flushed. The full thickness of the gastric wall is incised at the lesser curvature of the anterior wall of the gastric antrum to enter the abdominal cavity. After locating the gallbladder, the gallbladder wall is incised at the fundus. The bile is exhausted and the endoscope is inserted into the gallbladder cavity to treat the polyps and other lesions. The gallbladder wall incision is clamped with metal clips, after which the entrance of the gastric antrum is closed after fully flushing the abdominal cavity. (ii) Transrectal gallbladder-preserving polypectomy (TR-GPP): The balloon is placed in the transverse colon and inflated to block the colon lumen. The endoscope is inserted into the intestinal cavity through the anus and rectum, after which the intestinal cavity is fully rinsed and disinfected. Full-thickness of the intestinal wall is incised at the anterior wall of the rectum to enter the abdominal cavity. The operation is the same as TG-GPP after locating the gallbladder. The opening of the gallbladder wall is clamped with metal clips, the abdominal wall is fully washed, the incision of the bowel is clamped, and the air sac removed after puncturing.

In 2004, Kalloo et al. reported the first trial of transgastric natural orifice transluminal endoscopic surgery (NOTES) for abdominal exploration and liver biopsy on porcine models, confirming the technical feasibility and safety of the transgastric approach in the abdominal cavity [30]. In 2007, Marescaux et al. completed a transvaginal NOTES cholecystectomy in a patient with symptomatic cholelithiasis, which is considered to be the first clinical NOTES operation in the world [31]. Cases with transgastric access have been reported since then [32–34]. NOTES is minimally invasive and achieves a true “hidden scar” effect, which can meet the aesthetic requirements for some patients. However, all organs that can be preserved should not be removed, and the maximum preservation of organ functions is the real minimally invasive. Thus, NOTES cholecystectomy has not fully utilized the advantages of minimally invasive surgery. In 2015, Liu completed TR-GPP and gallstones extraction [35], which pioneered endoscopic gallbladder-preserving polypectomy procedures. Subsequently, TG-GPP also emerged [36, 37]. In a study that included 22 patients [37], 12 of whom underwent TG-GPP for polypoid lesions, four patients suffered localized peritonitis (4/22, 18.2%) after the procedure, which subsequently recovered after conservative medical treatment.

NOTEGPP has the advantage of less bleeding and significantly low adverse reactions, such as postoperative abdominal distension and incision pain. Compared to the transgastric approach, the rectal approach provides a more familiar view of the abdominal cavity for endoscopists who master LC and a clearer anatomical location of the upper abdominal organs. Nevertheless, preoperative preparation of the transgastric approach is convenient, there is no need to clean the intestinal tract, and the path is short, therefore, the operation time can be saved; the soft endoscope is used to operate in a curved state, thus, the position is relatively fixed.

### E-NOTEGPP

E-NOTEGPP is a surgical approach in which the gastroscope enters the abdominal cavity through a periumbilical incision, locates and dissects the gallbladder, and then it enters the gallbladder cavity to manage the polypoid lesions. He et al. found no obvious scar or recurrence in 12 patients with GBPs after surgery and 1-year follow-up, while gallbladder emptying functions were largely restored to preoperative levels [38].

Compared to NOTEGPP, this procedure opens access to the abdominal cavity from the body surface and is an almost scarless surgical method. Compared to Endolap polypectomy, E-NOTEGPP has the following advantages [38]: (i) The umbilical cord is the weakest part of the abdominal wall, and transumbilical puncture can reduce abdominal wall injury and relieve the pain from the surgical wound. (ii) The 2–4 incisions in the abdomen of the Endolap polypectomy can be transformed into a small incision of 10 mm in the umbilical cord. With few scars on the body surface after the operation, E-NOTEGPP achieves a minimally invasive and cosmetic effect.

### EUS-GC combined with PTEP

EUS-GC combined with PTEP is a combination of two procedures: EUS-GC, in which the gallbladder is scanned through the upper gastrointestinal tract (stomach or duodenum) under endoscopic ultrasound guidance and anastomosed to the wall of the upper gastrointestinal tract via placement of a stent, creating an artificial channel; PTEP, a surgical procedure for removing polyps after sinus tract formation and stent withdrawal, through the mouth, esophagus, stomach, duodenum and the formed sinus tract to the gallbladder.

In 2013, Mönkemüller et al. first reported a case of acute cholecystitis treated with EUS-GC combined with PTEP using a self-expanding metal stent [39], which provided a new treatment idea for patients with acute cholecystitis who have a need for biliary preservation or whose physical conditions are intolerant to surgery. This therapy has successfully been used for treatment of GBPs [40, 41]. Only one patient experienced severe peritonitis and complained of abdominal pain and fever following the cholecystostomy procedure, and was recovered after accepting anti-infective treatment [41].

Based on the anastomotic route, EUS-GC can be divided into gastro-gallbladder anastomosis and duodenum-gallbladder anastomosis. Regarding the former, the puncture point is selected in the gastric antrum while for the latter, it is selected in the duodenal bulb [42]. If there is duodenal ulcers and obstruction, gastro-gallbladder anastomosis should be chosen; if the patient has good duodenal morphology and functions, duodenal-gallbladder anastomosis is a better choice. This therapy is more in line with human physiology than PTCS: On one hand, the therapy majorly passes through the natural orifice of the human body; on the other hand, as an internal drainage scheme, EUS-GC avoids the loss of bile and the electrolyte disturbance it causes.

## Conclusion

As a well-developed procedure, LC has been used without hesitation by clinicians in the treatment of acute cholecystitis with gallbladder stones. Nevertheless, it has not been conclusively established whether resecting the gallbladder is the optimal therapeutic approach for GBP patients. Conventionally, “polyps ≥ 10 mm in diameter” is taken as an indication for cholecystectomy, however, the relationship between the diameter of GBPs and malignancy has yet to be conclusively determined. Blindly taking this as the criterion will increase the “misincision rate” of the gallbladder with benign lesions. Losing a functional gallbladder for benign polyps as well as delaying the diagnosis and treatment of GBC by insisting on gallbladder preservation are not ideal situations. Additionally, although complications of cholecystectomy have trended downward in recent years with the improvement of expertise, a 2011 analysis of 4113 patients who underwent LC for acute cholecystitis reported that 5.5% of patients had intraoperative complications and 6.1% had complications postoperatively [44]. The most common complications were abdominal wall or intra-abdominal bleeding (1.8%) and superficial wound infection (1.0%). Extrahepatic bile duct injury occurred in 17 patients (0.4%). The absence of the gallbladder is also associated with an increased risk of developing metabolic syndrome (OR = 1.872, 95% CI: 1.193–2.937) [45], nonalcoholic fatty liver disease (OR = 1.35, 95% CI: 1.03–1.77) [46], and irritable bowel syndrome (OR = 7.573, 95% CI: 1.096–52.318) [47]. In summary, in cases where the nature of the gallbladder polyps cannot be confirmed, the doctor should make a prudent decision to remove the gallbladder.

Guidelines issued by different associations have different recommendations on gallbladder polyps management. (Table 2) The guidelines only enumerate the risk factors, and clinicians need to combine their clinical experience to offer treatment advice to their patients. Thus, the doctors’ subjective experience plays a large part in decision-making. In contrast, the predictive models based on statistical methodology and AI, which visualize the risk level through objective numbers, are able to accurately assess the nature of polyps while preserving some gallbladders with benign polyps. The current predictive models reported in literature are limited by small sample sizes, small data types, and few study centers. International, multicenter, and large-scale clinical studies are required to establish more precise diagnostic models.

**Table 2:** Updated guidelines issued by different associations on the management of gallbladder polyps

Association(s)	Version	Guidance
Branch of Biliary Surgery, Chinese Surgical Society; Chinese Committee of Biliary Surgeons [50]	2019	Gallbladder polyps with malignant tendency have the following characteristics: (1) diameter ≥ 10 mm; (2) combined with gallstones or cholecystitis; (3) solitary or sessile polyps with a growth rate > 3 mm/ 6 months; (4) adenomatous polyps.
The Japanese Society of Hepato-Biliary-Pancreatic Surgery [51]	2019	For polypoid lesions of the gallbladder that are sessile, have diameters equal to or greater than 10 mm, and/or grow rapidly, prophylactic cholecystectomy should be performed.
ESGAR, EAES, EFISDS and ESGE [52]	2021	1. Cholecystectomy is recommended for: (1) polyps ≥ 10 mm in diameter as detected on transabdominal ultrasound; (2) polyps < 10 mm in diameter but with symptoms due to the gallbladder; (3) polyps between 6–9 mm in diameter and the patient has one or more risk factors such as age > 60 years, primary sclerosing cholangitis, asian ethnicity and sessile polypoid lesion (including focal gallbladder wall thickening > 4 mm). 2. If a patient has a risk factor, the presence of a solitary polyp strengthens the evidence that malignant potential exists, and cholecystectomy should be considered. 3. If during follow-up the gallbladder polypoid lesion reached 10 mm cholecystectomy is advised; if the polypoid lesion grows by 2 mm or more within the 2-year follow-up, its current size should be considered along with patient risk factors.
China Anti-cancer Association [53]	2022	1. For patients with gallbladder polypoid lesions who have clinical symptoms such as right upper abdominal discomfort after eating, if the polyps are ruled out as cholesterol crystals in the gallbladder through effective imaging examinations, or the symptoms are not relieved after choleretic treatment, cholecystectomy is recommended regardless of the size of lesion. 2. For asymptomatic gallbladder polypoid lesions, cholecystectomy is recommended if the following conditions exist: (1) the lesion is combined with gallstones; (2) the largest diameter of the lesion exceeds 10 mm (CT, MRI, endoscopic ultrasonography or contrast-enhanced ultrasound); (3) the base of lesion is wide; (4) the lesion is thin-stalked, intracapsular growth with good blood supply, and the polyp is clearly enhanced by enhanced CT examination; (5) the lesion is located in the neck of the gallbladder or near the opening of the cystic duct. 3. For those with asymptomatic gallbladder polypoid lesions who do not yet have indications for surgery, regular follow-up and examinations should be performed. Cholecystectomy is recommended when the following conditions are present: (1) age is over 50 years old; (2) the polyp has a maximum diameter of less than 8 mm, but compared with the imaging results (CT or MRI) within one year, it indicates that the lesion has grown significantly. (3) the polyp has a diameter of 6 mm, and enhanced CT examination shows that the blood supply is good.
Society of Radiologists in Ultrasound [54]	2022	If the gallbladder polyp seen on ultrasound does not meet exclusion criteria, its risk level can be determined by the morphology and the size. 1.Extremely low risk: pedunculated ball-on-the-wall or pedunculated with thin stalk (1) ≤ 9 mm: no follow-up; (2) 10–14 mm: follow-up ultrasound at 6,12,24 months; (3) ≥ 15 mm: surgical consult. 2.Low risk: pedunculated with thick or wide stalk or sessile (1) ≤ 6 mm: no follow-up; (2) 7–9 mm: follow-up ultrasound at 12 months; (3) 10–14 mm: follow-up ultrasound at 6,12,24,36 months vs. surgical consult; (4) ≥ 15 mm: surgical consult. 3.Indeterminate risk: focal wall thickening ≥ 4 mm adjacent to polyp (1) ≤ 6 mm: follow-up ultrasound at 6,12,24,36 months vs. surgical consult; (2) ≥ 7 mm: surgical consult.

ESGAR, European Society of Gastrointestinal and Abdominal Radiology; EAES, European Association for Endoscopic Surgery and other Interventional Techniques; EFISDS, International Society of Digestive Surgery -European Federation; ESGE, European Society of Gastrointestinal Endoscopy; CT, computed tomography; MRI, magnetic resonance imaging

Conclusions reached with assistance of predictive models are still less accurate than pathological examinations. The gallbladder-preserving polypectomy procedures allow for treatment of gallbladder polyps while providing a diagnostic service. The removed polyps are sent for pathology, and the gallbladder is preserved for benign cases, while malignant cases are further treated by cholecystectomy, thereby eliminating the missing detection of GBC and reasonably preserving gallbladder functions. As a third option other than “to resect” or “not to resect “, the gallbladder-preserving polypectomy procedure is rarely mentioned in the above guidelines.

With reference to the 2021 edition of the guideline published by the Gallbladder-Preserving Surgery Committee of Endoscopy Specialist Branch of Chinese Medical Doctor Association [24], coupled with literature reports on minimally invasive gallbladder-preserving polypectomy procedures, the indications for gallbladder preservation are: (i) Patients with imaging findings that are suggestive of gallbladder polypoid lesions without malignant signs and with good gallbladder emptying functions. Mild chronic cholecystitis or gallbladder stones can be combined; (ii) GBPs > 5 mm in maximum diameter; (iii) Gallbladder wall thickness ≤ 5 mm and (iv) Patients with a desire for gallbladder preservation. Gallbladder preservation is not recommended if: (i) There are preoperative signs indicating: acute cholecystitis combined with purulence, gallbladder perforation and gangrene, yellow granulomatous cholecystitis; chronic cholecystitis with uniform thickening of the gallbladder wall > 5 mm or uneven thickness of the gallbladder wall; porcelain gallbladder; diffuse adenomyosis of the gallbladder; preoperative imaging suggesting the possibility of malignant gallbladder tumors; (ii) Intraoperative conversion to cholecystectomy is required if the following signs are found: gallbladder atrophy, intraoperative confirmation of the disappearance of gallbladder lumen, or too small volume; gallbladder duct obstruction, which cannot be intraoperatively released; intraoperative suspicion of malignant gallbladder tumors; intraoperative frozen pathology suggesting malignant gallbladder tumors or high-grade intraepithelial neoplasia, or uncertainty of benign lesions; (iii) Lesions that are benign on intraoperative frozen pathology but confirmed to be malignant on postoperative paraffin pathology require second-stage radical cholecystectomy.

To assess the treatment effect of gallbladder-preserving therapy, the methods of testing the gallbladder function have to be mentioned. Currently, gallbladder-emptying function is assessed clinically by comparing the variation of cholecyst volume between fasting and high-fat diet with the help of ultrasound [27, 38, 41]. This approach approximately treats the irregularly shaped gallbladder as a regular ellipsoid [48]. Although its accuracy needs to be improved, it is ideally suited to the clinic because it is non-invasive and easy to perform. Recently, a model of gallbladder motility has also been proposed and preliminarily validated [49], though neither the model nor the ultrasound method has been validated in clinical trials, and subsequently has not gained wide acceptance. Therefore, new tests, which can also be correlated with clinical symptoms, are needed to help assess gallbladder function more accurately.

There are concerns that postoperative gallbladder emptying functions may be affected, therefore, the importance of strict surgical indications should be emphasized. Doctors should preoperatively assess whether the gallbladder is functional or not based on clinical experience and patients’ examination indices. Moreover, they should evaluate the feasibility of gallbladder preservation according to what they see intraoperatively. It has been demonstrated that gallbladder emptying functions return to preoperative levels 1 year after surgery and devices to close the gallbladder wound (such as hemostatic clips) do not provoke discomforts in patients or affect gallbladder functions [38].

With advances in medical technologies, the routes for gallbladder-preserving polypectomy procedures have evolved from “transabdominal” to the more physiological routes, through the natural cavity of the body. The procedures are able to preserve gallbladder functions while maintaining the cosmetic effects of no scars. The departments that perform gallbladder-preserving polypectomy procedures have expanded to interventional medicine, hepatobiliary surgery and gastroenterology. However, evidence for applications these therapies, especially NOTEGPP and EUS-GC combined with PTEP, is limited by the small sample size and unknown long-term efficacy, for which relevant large-scale clinical studies are required. Meanwhile, researchers should also focus on comparing gallbladder-preserving procedures with mainstream LC in a comprehensive manner, encompassing procedure-related factors such as adverse event rates and haemorrhage, patient-related factors such as postoperative pain assessment, and economic factors such as hospitalisation costs. Compared with LC, some patients undergoing gallbladder-preserving procedures will inevitably encounter the recurrence of GBPs. By following up these patients, researchers can explore the risk factors for polyp recurrence and whether changing the diet and taking choleretic drugs can reduce the recurrence rate, which is significant for improving the gallbladder-preserving treatment.

In conclusion, establishment of predictive models and the rise of gallbladder-preserving polypectomy procedures in recent years have placed an increasing emphasis on the role of the gallbladder and inform on when to perform cholecystectomy. By reviewing the progress in management of GBPs in the past decade, we hope that clinicians will work together to further explore and improve the relevant techniques and therapeutic indications.

## Data Availability

Not applicable.

## Abbreviations

AI: Artificial intelligence
CI: Confidence interval
CSA: Cross-section area
CT: Computed tomography
E-NOTEGPP: Embryonic-natural orifice transumbilical endoscopic gallbladder-preserving polypectomy
Endolap: Endoscopic-laparoscopic
EUS-GC: Endoscopic ultrasound-guided cholecystostomy
GBC: Gallbladder cancer
GBP: Gallbladder polyp
LC: Laparoscopic cholecystectomy
La-CGPP: Laparoscopy-assisted choledochoscopic gallbladder preserving polypectomy
M-CGPP: Minilaparotomy choledochoscopic gallbladder preserving polypectomy
NOTEGPP: Natural orifice transluminal endoscopic gallbladder-preserving polypectomy
NOTES: Natural orifice transluminal endoscopic surgery
OR: Odds ratio
PCP: Percutaneous cholecystoscopic polypectomy
PTCS: Percutaneous transhepatic cholecystoscopy
PTEP: Per-oral transmural endoscopic polypectomy
TG-GPP: Transgastric gallbladder-preserving polypectomy
TR-GPP: Transrectal gallbladder-preserving polypectomy
